# Extra Supplement—The Nursing Mirror

**Published:** 1890-07-19

**Authors:** 


### "The Hospital" Nursing Mirror.

Being the Extra Nursing Supplement of "The Hospital" Newspaper.

All Contributions for this Supplement should be addressed to the Editor, The Hospital, 140, Strand, London, W.C., and should have the word "Nursing" plainly written in left-hand top corner of the envelope.

### En Passant.

Wolverhampton Institution.—The annual report of this Queen Victoria Nursing Institution comes to us in a terribly brilliant blue cover. With most of the facts it gives we dealt at the time of the annual meeting, but we are glad to learn more fully how thoroughly successful the Home is, and how much the work of the lady superintendent and the nurses is appreciated.

Torbay Hospital.—Through the thoughtful kindness of the chairman, one of the committee, and two of the medical staff of this hospital, the nurses have been able to enjoy a coach drive through the most beautiful scenery in Devonshire. Could those with money only realize the intense pleasure a day off gives a nurse, who must necessarily spend the greatest part of her life in sick rooms, we feel sure there would be many imitators of this plan.

Philadelphia School.—A nurse writes to us from Philadelphia to complain of an accidental mistake in our article of May 31st, on "American Training Schools." We stated that the Boston City Hospital had the largest training school in the States. This is not so; Boston has only sixty-seven pupils, to one hundred pupils at the Philadelphia Hospital. We are grateful to our correspondent for the correction and her kind letter; we are only too pleased to hear of the grand success of the work started by the late Miss Alice Fisher.

Short Items.—The annual meeting was held at Marylebone Infirmary on the last day of June. A large number of visitors turned up to tea. The probationers were complimented on their examination papers, though the number of marks were not so high as last year.—A branch of the Workhouse Infirmary Nursing Association is to be shortly started in the North of England.—Eight Red Cross nuns from Kingwilliamstown Convent have gone to Bechuanaland to establish a hospital in some suitable place for the new police force and the African Company's employés.—The Huddersfield Nurses' Home has been started by Miss Murray, at 58, Westfield, Trinity Street.—A nurse writes to ask us if it is fair to stow nurses' boxes in a damp cellar? Certainly not.—It is Sister Mark, of Bart.'s, who signs the appeal of the Santa Claus Society.

The Hospital Commission.—The proceedings before Lord Sandhurst have lately referred almost exclusively to nursing, and have excited such interest in nursing circles, we feel bound to refer to them here, though no criticism can fairly be offered till the Commission issues its report at the close of the sittings. The room in the House of Lords where the Committee sits has lately been unpleasantly crowded with nurses, and the matrons of most of the London hospitals were present. The evidence has referred especially to the nursing at the London Hospital, which received a bad character from some old probationers. At the last meetings, however, the other side have had their story to tell, and when the matron read from her private register her view of the characters of these old probationers some of them must have had a foretaste of the judgment day. It is worthy of note that the nurses of the London Hospital have signed a memorial expressing sympathy with their matron.

Workhouse Nurses.—If any strong, sensible woman, under forty, and with a year's experience of hospital or infirmary nursing, is anxious to spend and be spent in the cause of the suffering poor, she cannot do better than join the Workhouse Infirmary Nursing Association. There she will find work wanting all the powers a woman can give—work which needs tact and strength, and time and skill. Boards of Guardians will refuse her necessaries, pauper patients will grumble (or worse) at her ministrations, and trials and privations will meet her at every step. She may be given work near the London Docks, or away in a dull country union, but wherever it is it will be difficult. What is good is always difficult, says Aristotle, and there are women who have the courage and the patience to accept posts like these, but at present there is a dearth of them, and the association pleads for more nurses and more funds. The monetary reward of the nurse may be only £20, but the content gained by successful achievement, the knowledge of personal worth, is an enduring gratification which should brighten any woman's life.

Nightingale Nurses.—The report of the Nightingale Fund, just issued, tells of seventy-one nurses in training last year. Twenty-six probationer-nurses were entered on the register of certified nurses, after satisfactorily completing their year of training, and of these eight were special probationers, and eighteen nurse probationers. The annual gratuity of £2 allowed by the regulations for the term of three years to the certified nurses who have completed a year's service satisfactorily in some approved hospital or other institution for the benefit of the sick poor, was awarded to sixty-nine nurses, viz.: Nineteen for their third year, twenty-three for their second year, and twenty-seven for their first year. Of the twenty-six probationers who completed their training twenty-four received appointments as follows, viz.: St. Thomas's Hospital, two first as nurses, then as ward-sisters, one night nurse, two day nurses, sixteen extra nurses and one assistant in the Nightingale Home; Metropolitan and National Nursing Association, Bloomsbury Square, one nurse; St. Marylebone Infirmary, one day nurse; and Royal Infirmary, Edinburgh, assistant night superintendent. During the year forty-eight lectures were given by the hospital professors, of which fourteen were of a clinical character; Dr. Bristowe gave several clinical lectures, but having to alter the time of giving his usual course, Dr. Bernays was so good as to deliver two courses to different sets of probationers on chemistry and hygienic subjects, which were followed by examinations, with the result of which Dr. Bernays expressed himself much satisfied. Mr. Croft gave ten lectures on anatomical subjects, followed by an examination; and eleven on clinical subjects. He also held quarterly examinations, as usual, of probationers in their fourth quarter, and was much gratified with the results; twenty-seven probationers were passed. At the Marylebone Infirmary Miss Vincent states that fifteen probationers were trained and all received on the staff. Mr. Lunn and Miss Moriarty are thanked for their help in training these probationers. It is impossible to read this report and not appreciate the fact that the Nightingale nurses are lucky nurses.

### Sketches of Insanity.

By Francis H. Walmsley, M.D. (Senior Assistant Medical Officer of Leavesden Asylum).

### III.—THE FALSE BELIEFS OF THE INSANE.

"O, hateful error, melancholy's child !
Why dost thou show to the apt thoughts of men
The things that are not !"—Julius Cæsar.

"I talk of dreams,
Which are the children of an idle brain,
Begot of nothing but vain fantasy,
Which is as thin of substance as the air."
—Romeo and Juliet.

The following graphic description, written by one who had recovered from an attack of mental derangement, is highly instructive, and affords us an insight into the working of the disordered brain.

"My senses were all mocked at and deceived ; in reading, my eyes saw words on the paper which, when I looked again, were not. The forms of those around me, their features, changed even as I looked on them. I heard the voices of invisible agents, and notes so divine, so pure, so holy, that they alone perhaps might recompense me for many sufferings ; my sense of feeling was not the same, my smell, my taste was gone or confounded."

The phenomena of dreaming have a striking analogy or likeness to the phenomena of some forms of unsound mind. When asleep we can experience nearly all the episodes which meet us in lunatic asylums. All men while they are awake are in one common world, but each of them when he is asleep is in a world of his own ; the lunatic has no common world, he lives and moves in a private world that is particular to himself—a wide realm of wild reality.

By bringing vividly before our minds the scenes and incidents in which we played a part during one of our short night-dreams, we shall the better understand the long day-dream of the insane, and read aright the thoughts and passions, the tears and tortures, and the joys by which they are possessed.

*The Errors of the Understanding* are known as Hallucinations, Illusions, and Delusions.

Hallucinations and Illusions are disorders of perception, and are related to the special senses of sight, hearing, touch, smell, taste, and the muscular sense. Delusions are disorders of the reasoning power.

*Hallucination* : A diseased state of mind in which a person has a settled belief in the reality of things which have no existence, as when a man in total darkness thinks he sees an object ; the *blind* man may see visions and the *deaf* man hear sounds. A person suffering from hallucinations neither sees, nor hears, nor feels, nor smells, nor tastes anything in reality, though he believes that he does so ; thus we see that hallucinations are *subjective*, or arise solely within the brain.

*Illusion* means a mockery, false show, or counterfeit appearance—as when a person sees, or hears, or smells, or tastes, or touches something, and takes it to be something else ; or when a man under the influence of terror takes a stump of a tree, whitened by the moon's rays, for a ghost.

*Delusions* are erroneous or false judgments formed by the mind, as distinguished from false perceptions ; an illusion of the senses if believed to be a reality becomes a delusion of the mind.

A man may have delusions regarding himself, his friends, his profession or occupation, and his worldly circumstances.

Delusions cannot be corrected by argument or experience.

*False Perceptions of the Sane:* Illusions are frequently observed in a state of mental health, being then corrected by the reason.

Byron imagined himself to be sometimes visited by a spectre ; but he said it was due to over-excitability of his brain.

Doctor Johnson heard his mother call "Samuel," though she was then living in a distant town.

Swedenborg, as he advanced in years, believed that he was an abnormal person, to whom was granted the privilege of conversing with angels and spirits.

Joan of Arc was continually urged by voices to deliver her country from the invader.

An Individual meeting a friend in the street could not tell at once whether it was a phantasm he saw or an actual person, until he heard the footfalls. Napoleon the Great had his "star." "I see it," said he, "in every occurrence ; it urges me onward, and is an unfailing omen of success."

Reference may here be made to the remarkable phenomenon known as "colour-bearing," in which, with some musicians, different notes have their corresponding colours.

Examples of False Beliefs of the Insane.—A patient hears voices speaking to him which no one else can hear, or sees objects which no one else can discover. He hears commands from heaven or threats from evil spirits ; he converses with people under the floor.

Patients suffering from lowered sensibility of the organism imagine that they have lost their personality ; that they are changed into animals ; that they have become inanimate things, as lumps of clay, glass, or butter, or even that they are no longer alive.

In other individuals, owing to the abnormal information they receive from different regions of the organism, the personality becomes raised several degrees above its normal pitch ; a person becomes richer, greater, stronger ; he speaks of himself, of his physical strength, which is immense ; of his social importance, which is enormous ; he has become a King, Emperor, Pope, &c.

Patients suffering from derangements of taste or smell are tormented by the thought that attempts are being made to poison them.

A female patient fixes a postage stamp on her forehead, and believes she goes through the pillar post long journeys to visit her relatives ; the following morning she affixes another stamp and is returned, through the post, to the asylum.

An individual believes that he is a bad half-crown, and will go round to the neighbours warning them not to take him in payment or to give change for him when his wife offers him at the counter.

A sempstress working late at night impaired her eyesight ; she saw at the same time four hands, four needles, and four seams. She at first treated this as an illusion, but at the end of some days, in consequence of weakness and prolonged mental anxiety, she imagined that she was really sewing four seams at once, and that God, touched by her misfortunes, had worked a miracle in her favour.

A deeper knowledge of the working of the unsound mind, and a more perfect understanding of the sayings and the doings of the insane will teach us that we must regard them not as *obstinate* and *self-willed* (terms which should never be heard in a lunatic asylum), but as beings influenced and dominated by trains of thought which are, to them, as reasonable and logical as are our own ; and thus, by reason of our improved methods, we may hope for a greater measure of success in our endeavours to lift

"The cloud, which, intercepting the clear light,
Hangs o'er the eyes and blunts the mortal sense."

(*To be continued.*)

### An Appeal to the Humanity of the Public.

In this far-away land of Australia I have but lately received, and not ten minutes since finished reading, in a number of The Hospital for December 14th, 1889, the appeal of "A.E.S." to its readers on a subject that I firmly trust will before long address itself to all advocates of justice and common sense in both hemispheres. It is made on behalf of members of a profession to which I have the honour to belong, and deals with a subject I have, ever since I understood anything of it, intended some day to take up and ventilate to the best of my ability. I have only waited for time and sufficient standing in my calling to justify my dealing with the matter, before addressing the public with a view to persuading them to do away with a standing reproach to common sense and humanity. I allude to the long working hours of hospital nurses, for we are no better off in this respect than our fellow-workers in England and elsewhere. "A.E.S." writes in the weary, hopeless tone of an overworked, and consequently depressed and pessimistic woman, and I feel acutely for her. I have been in that mood so often myself, and from the same cause. I love my profession, and shall stick to it as long as I have any capacity for work left in me, but at the rate I have been going for the last five years that will not be for long. During that period force of circumstances and interest in work that wanted doing, added to hospital regulation, have made me cram as much work into 24 hours as should have had 72 for its performance, or three women to do it. As trainer at the Alfred Hospital, Melbourne, I worked hard in charge of 70 beds. At another large hospital in the same city I worked harder for some years—so hard that I could not have gone on much longer at the pressure, and hundreds of women can give you the same experience. Now, as matron of an up-country hospital, I am recruiting and gathering energy for a fresh start, I hope, as matron to a large hospital, with management of a training school for nurses, and it is with this ambition I have trained and made a study of hospital life and hospital nurses, and with this in view I have thought long and often of its regulations and their overwork. The evils are very evident, the remedies difficult to find, but they have been found for other workers in the world, and are being found for more and more of them as time goes on, and the day must be coming when hospital nurses will have the eight hours' work, eight hours' recreation, and eight hours' sleep that strong men find they cannot do without. We cannot leave our work as can the artisan or labourer, but why cannot we have shifts of workers as do the miners?

Consider for a moment what a hospital nurse's day is. On duty at 6 a.m. entailing a rise at 5.30 every day in the year, exposed to contagion and poison in varied forms, and hard at work physically and mentally till 6 and 8 p.m., barely able to take the three half-hours allowed in most institutions for meals. In some hospitals the nurses get an afternoon off duty from 2 to 10 p.m. once a week, one Sunday from 2 to 10 each month, and fourteen days' holiday in the year, but these rules do not exist by any means in all. I am quoting from a fairly large knowledge of Australian Hospitals, and I do not think our sisters in England are any better off, and for such work and such hours at home, nurses' pay varies from £20 to £60 per annum; in Australia from £20 to £80 per annum. And we are not ignorant and rough women nowadays, but mostly of gentle rearing, education, and refinement, learning our profession by all the study we can make time for after our fourteen hours' practical work in the wards is over, having to pass severe examinations if we would render ourselves up to the mark and gain such certificates for skilled nursing as will give us a standing in the nursing world and enable us to earn a living. Can you name any of the world's work of a more trying and exhausting nature than a hospital nurse's? For long hours day after day, women, the weaker vessels, are breathing vitiated air, witnessing harrowing sights, doing work that motive only keeps from being revolting, carrying on their shoulders an amount of responsibility that wears them out—for we are most of us intensely interested in our patients, and understanding their condition and the objects of the treatment—keenly appreciative of our responsible office, well knowing that in the majority of cases a mistake or any half-hearted work may be fatal, having to bear in mind and accurately carry out all the numerous and difficult orders dictated by the medical and surgical skill of these scientific days. The science of healing acknowledges nursing as more than its handmaid now, recognising in her a sister and valuable coadjutor, without whose aid doctors may work in vain. The excuse for overworking nurses cannot hold good now, that though their hours are long their responsibility is small. Doctors well know that the responsibility is well nigh equal, though the reputation is not so much at stake. Yet woman, with her weaker body and smaller brain power, has to exercise them for twelve and fourteen hours daily, all the while keeping cheery, patient, light-handed, and skilful, and by her appearance and manner, besides her actual work (and they are part of it), helping on her patient's recovery. Are you not requiring of us women superhuman exertions? Are many surroundings more calculated to sap energy and shorten life than a hospital nurse's? Do not we pay a heavy tax to death yearly—a tax that might be lightened and the strength of a most useful community husbanded by giving to its members the same length of working hours that men have? We have the satisfaction of good work well done in our hearts, a life spent in a calling that embraces no element of humbug. No money pays for all we do, but will you not plead for some time for us to appreciate the brighter side of life and by a certain amount of leisure and pleasure render us fit to work, if not better, for much longer?

I have badly pleaded a good cause, so many thoughts on the subject rush through my mind. There is so much to advance in one cause, it is difficult for me to condense it well in this, my first appeal, to a just and generous public, who have but to recognise a wrong to endeavour to get it righted. Do not, therefore, be heedless of the pleading of one who has tried and knows the cost of twelve and fourteen hours' hard physical and mental strain on a woman.

A. M. M.

### The Nurses' Bookshelf.

### The Registration of Nurses.*

This small pamphlet is a summary of the case against registration, being a reprint of the Memorial of the Nurse-Training Schools, the discussion thereon, and the resolutions adopted by the General Council of Medical Education and Registration. It is a pamphlet which has a decided value of its own, especially at the present time when the Lords' Committee is dealing with the question of nurses and nursing. Of course, only one side of the question is shown, but the reasons for a ripe scheme of registration are fairly given, while the faults of the present scheme are fully set forth. Matrons who desire to put this view of the question before their nurses cannot do better than procure 100 copies of this pamphlet for distribution.

(* The Registration of Nurses. Published by "The Hospital" (Limited), 140, Strand, W.C. Price 3d. each, or 1s. per doz., 5s. per 100.)

Thrift is being encouraged among the lower classes in Paris by the promotors of the "Sou Quotidien." The putting by of a daily sou regularly for fifteen years is to be requited by a regular allowance at the end of that period.

### Everybody's Opinion.

[*Correspondence on all subjects is invited, but we cannot in any way be responsible for the opinions expressed by our correspondents. No communications can be entertained if the name and address of the correspondent is not given, or unless one side of the paper only be written on.*]

### ONCE IN A LIFETIME.

"One of the Thousand," writing from Philadelphia, U.S.A., says: I am looking forward to the time when the ever-welcome Hospital will come with an account of the presentation by the Princess at Marlborough House. I am among the first 300 of the first 1,000 nurses who are invited to receive their certificates on that occasion, and as it is one of those interesting things that occur but once in one's lifetime, perhaps some of my fellow nurses can understand with what reluctance I was compelled to send a non-acceptance to the invitation forwarded to me by the kind and indefatigable secretary of the Pension Fund. I fully intended spending my first holiday for three years at home in dear old England, but am compelled to make "my destiny my choice" and remain with an "obstinate case of melancholia" with whom I have now spent one year.

We are spending a few weeks by the "sad sea waves" at Cape May, New Jersey. The Americans claim that the beach here is one of the finest in the world, and it is fine; but I am longing for an English beach, and English things, and home. Time and patience will bring, or rather take, me to all these, and I must feel thankful for the health and strength I have been blessed with during the time I have spent here. The good kind physician whose patient I am nursing (Dr. Weir Mitchell) sent me such a kind encouraging letter before he left the city for his annual holiday. He will not return till November, as he never does any work after June, and we are all very glad when the "fall" brings him back to us.

### A PLEA FOR CHEAP TICKETS.

Nurse Webster writes:—Now that the summer (and with it the holidays) is coming on apace would it not be possible to try and induce the managers of the different railway companies to grant reduced fares for the double journey, to such nurses who cared to apply? Many nurses have friends who live at long distances, and the return fare costs something like £2 or £3, this, out of a nurse's scanty earnings, is a great consideration. Trusting you will give the matter your consideration in the pages of The Hospital.

### A MODERN MIRACLE.

"Franciscus" writes:—Allow me to call attention to a few points in your article on "A Modern Miracle. (1) The authoritative voice of the doctor said "it is croup." In this the doctor erred. But the non-professional mind accepts with becoming humility the professional opinion. The ignorance of a medical man, and not the credulity of a Franciscan friar, is the cause of the blunder. For (2) had the child been near suffocation from croup—in such danger as to oblige a doctor to run for his instruments in order to perform tracheotomy—the expulsion of the membrane immediately on the swallowing of a spoonful of water would have been a very unusual termination of an attack. Such a result following promptly on such a remedy administered with the expectation that it would effect a cure of an imminently dangerous disease, would certainly justify the patient in quoting the circumstance in a discussion on the efficacy of "Lourdes Water" as a remedy. Strictly speaking, as a proof, he could not have intended it. I believe authenticating a miracle is almost as difficult and slow a business as encouraging a saint, and an unauthenticated miracle, cannot be offered as a proof. An extraordinary coincidence may fairly be offered as evidence all the same! (3) Anyhow the child was in danger of death or tracheotomy. Had the mother known the facts she would probably have clapped him on the back, or put her fingers down his throat the while she said a fervent prayer, knowing that miracles are not to be looked for where ordinary remedies suffice. As the case was represented to her, however, it was desperate, and she at once applied a supernatural remedy. Under the circumstances the supernatural remedy cured by a natural result. Faith in Our Lady was the cause; consecrated (or Lourdes?) water the means; preservation from immediate death, or a very dangerous operation the result. Lastly, if the coughing up of the skin was not the natural result of drinking the water, which seems most unlikely considering the sequence of events, the immediate cure of the child at the instant when the mother sought the help of Our Lady was a remarkable coincidence. The summing up of the case seems to be that the doctor, and not the friar, is to be blamed for offering this event as evidence of a cure worked by supernatural means, and that the doctor's mistake arose from want of knowledge and not from credulity. Moreover, that even as the case stands, the recollection of it might well incline any mother under similar circumstances to invoke the aid of "Our Lady of Perpetual Succour." Those who believe that Our Lord began His public ministry before "His hour was come" at the unspoken desire of His mother, will not doubt the efficacy of her intercession now.

### Notes and Queries.

### Answers.

*B. N. A.*—Not so. We are not so narrow-minded as that. We received a private card but not a press card, and so, of course, did not report the proceedings.

*Lady Nurse.*—Please send name and address, and we will publish your letter.

*A. C. G.*—Apply at the London Hospital, Whitechapel; all creeds are admitted there.

*Nurse S. A. M.*—Everyone has been thanked quite enough; we are glad you are grateful and that you enjoyed yourself. The brown case in which the certificate was presented is the safest.

*Uniform.*—When a nursing institution advertises "all uniform provided, but must be returned on leaving" it ought not to charge you for the making up of the uniform. At some institutions a small sum (3s. 6d. for instance), is allowed the nurses for making up the dress and the same for the cloaks. It is certainly not the custom to charge the nurse.

### Vacancies.

The following vacancies are announced:

### Matrons.

Dinorwic Hospital, Wales.

Kent County Asylum (Ass.); £85.

Tunbridge Wells Hospital; £50.

Manchester Maternity Hospital; £50.

### Nurses.

Aberdeen Poorhouse; £22.

Barony Hospital, Glasgow; £24.

Barton Regis Union; £20.

Beckett Hospital, Barnsley; £20.

Birmingham Eye Hospital; £25.

Bishops Stortford Home; £25.

Blackheath Nursing Institution.

Brighton Borough Hospital; £24.

Brighton Children's Hospital; £25.

Cardington Children's Hospital.

Chelsea Infirmary; £18.

Cheltenham Nursing Institute.

Cumberland Infirmary (Night); £22.

Frome Nurses' Home; £22.

Glamorgan Infirmary; £24.

Greenwich Union (Head); £27.

Greenwich Union; £20.

Hanover Institute; £25.

Huddersfield Infirmary (Night); £24 12s.

Huddersfield Nurses' Home.

Jersey Institute,; £25.

Manchester Sick Poor Nursing Institute.

Middlesbrough Fever Hospital; £30.

Mile End Old Town Infirmary; £17.

Mr. Wilson's Nursing Institution.

Newcastle Infectious Hospital; £30.

New Winchester Union; £20.

Northern Fever Hospital; £22.

North-Western Fever Hospital; £30.

Nurses' Home, Bengeo, Herts; £30.

Nurses' Home, Newcastle-on-Tyne.

Rotherham Hospital; £16.

Royal Chest Hospital; £22.

Sheffield Nurses' Home.

Sheffield Union Infirmary; £20.

Shoreditch Infirmary; £20.

St. Cuthbert's Poorhouse, Edinburgh; £25.

St. Mark's Hospital; £30.

St. Peter's Hospital, Covent Garden; £24.

St. Saviour's Infirmary, S.E.; £20.

Surrey County Asylum.

Swansea Institute; £25.

Windsor Infirmary; £20.

Workhouse Infirmary Nursing Association.

### District Nurses.

Hulme Nurses' Home.

Jersey Institute; £25.

Nottingham and Notts Nursing Association (Supt.); £60.

The Bank, Boston.

Tiverton.

Trinity Vicarage, Derby: £50.

### Probationers.

Arbroath Infirmary.

Bedford Infirmary.

Hammerwich Cottage Hospital.

Huddersfield Infirmary; £12 12s.

Kidderminster Infirmary.

Kilmarnock Infirmary.

Leeds Nurses' Institute; £14.

Lincoln County Hospital.

Preston Royal Infirmary.

Stamford Infirmary.

Wigan Albert Edward Infirmary.

### Amusements and Relaxation.

### SECOND QUARTERLY WORD COMPETITION.

Commenced July 5th, 1890, ends Sept. 27th, 1890.

Three prizes of 15s., 10s., 5s., will be given for the largest number of words derived from the words set for dissection.

Proper names, abbreviations, foreign words, words of less than four letters, and repetitions are barred; plurals, and past and present participles of verbs, are allowed. Nuttall's *Standard* dictionary only to be used.

N.B.—Word dissections must be sent in WEEKLY not later than the first post on Thursday to the *Prize Editor*, 140, Strand, W.C., arranged alphabetically, with correct total affixed.

The word for dissection for this, the THIRD week of the quarter, being

"CURRANTS."

| Names. | July 10th. | Totals. | Names. | July 10th. | Totals. |
|---|---|---|---|---|---|
| Dulcamara | 32 | 32 | Nurse Hilda | 29 | 29 |
| Tinie | 32 | 32 | Lightowlers | 28 | 28 |
| Henri | 31 | 31 | Jenny Wren | 25 | 25 |
| Agamemnon | 31 | 31 | Rhoda | 22 | 22 |
| Patience | 30 | 30 | Annabel | 17 | 17 |
| Brisbane | 28 | 28 | | | |

### Notice to Correspondents.

N.B.—Each paper must be signed by the author with his or her real name and address. A *nom de plume* may be added if the writer does not desire to be referred to by us by his real name. In the case of all prize-winners, however, the real name and address will be published.

Competitors can enter for *all* quarterly competitions, but no competitor may take more than *one first prize* during the year.

